# Psychometric evaluation of the Chinese version of the Patient Self-Advocacy Scale using classical test theory and item response theory

**DOI:** 10.1038/s41598-025-91129-2

**Published:** 2025-02-26

**Authors:** Jialu Cui, Jing Wang, Ailin Yue, Jiayan Cao, Zhijie Zhang, Baoxin Shi

**Affiliations:** 1 Hospice Care Research Center, The School of Basic Medical Sciences, Tianjin Medical University, Tianjin, China; 2 Clinical College of Ophthalmology, Tianjin Eye Hospital, Tianjin Medical University, Tianjin, China; 3 Tianjin Medical University Cancer Institute and Hospital, Key Laboratory of Cancer Prevention and Therapy, National Clinical Research Center for Cancer, Tianjin’s Clinical Research Center for Cancer, Tianjin, 300060 China

**Keywords:** Patient advocacy, Psychometrics, Head and neck neoplasms, Quality of life, Rasch analysis, Psychology, Health care, Medical research, Oncology

## Abstract

Background: Patient self-advocacy plays a crucial role in improving cancer patients’ quality of life, but there is no validated instrument to assess this concept among Chinese head and neck cancer patients. This study aimed to cross-culturally translate the Patient Self-Advocacy Scale (PSAS) and evaluate its psychometric properties using classical test theory and item response theory. Methods: The PSAS underwent cross-cultural adaptation based on Brislin’s translation model and a cross-sectional survey of 302 head and neck cancer patients at a tertiary hospital in Tianjin was conducted from November 2023 to August 2024. Classical test theory was used for item analysis and validation of reliability (internal consistency, test-retest reliability) and validity (content validity, construct validity). Item response theory was applied to evaluate model fit, reliability, item difficulty, and measurement invariance. Results: Classical test theory analysis demonstrated good item discrimination with item-total correlations ranging from 0.776 to 0.942 and critical ratios from 13.269 to 33.170 (*p* < 0.05), as well as good internal consistency (Cronbach’s α = 0.942 for the total scale) and test-retest reliability (ICC = 0.840 for the total scale, *p* < 0.001). I-CVI values ranged from 0.80 to 1.00, with an S-CVI of 0.95. The three-factor model demonstrated good fit (χ^2^/df = 2.595, RMSEA = 0.090, SRMR = 0.072, CFI = 0.966, IFI = 0.966, TLI = 0.956). Rasch analysis indicated a good model fit and reliability (person/item separation index > 1.5, person/item reliability coefficient > 0.9). The Wright map showed good matching between item difficulty and person ability. Differential item functioning (DIF) analysis revealed no significant differences across gender. Conclusion: The Chinese version of PSAS demonstrates satisfactory psychometric properties among head and neck cancer patients and provides healthcare providers with a tool to assess patients’ self-advocacy, potentially facilitating patient-centered care and self-management in clinical practice and improving patients’ health and quality of life outcomes.

## Introduction

Head and neck cancer (HNC), encompassing malignancies in the upper aerodigestive tract^1^, represents the sixth most common cancer type globally, with GLOBOCAN 2022 data indicating 947,211 annual cases accounting for 4.74% of global cancer incidence^2^. In China, the HNC burden has shown a consistent increase, with new cases rising from 135,100 in 2015 to 145,600 in 2022, constituting 15.37% of global cases^3,4^. The therapeutic modalities for HNC encompass chemotherapy, radiotherapy, surgery, and immunotherapy-based approaches, which can be administered either as monotherapy or in various combinations^1,5^. While advances in contemporary therapeutic modalities have markedly improved five-year survival rates among HNC patients^6,7^, these improvements have introduced novel challenges regarding patients’ quality of life. Due to the unique anatomical complexity of the head and neck region, therapeutic interventions inevitably impact critical functions, including respiration, deglutition, and verbal communication. HNC survivors frequently experience multiple symptom burdens, including xerostomia, pain, and fatigue, while simultaneously confronting challenges related to altered body image^8^, compromised intimate relationships^9^, and occupational reintegration difficulties^10^. These challenges often precipitate psychological distress, manifesting as anxiety, depression, and suffering^11,12^. Collectively, these treatment-associated toxicities have profound implications, substantially impacting the quality of life^13^, diminishing treatment adherence^14^, and potentially compromising survival outcomes.

Current interventional strategies aimed at enhancing the quality of life among HNC survivors predominantly emphasize external support mechanisms, such as health education and symptom surveillance^15,16^, while insufficient attention is given to patient autonomy and active engagement^17^. In contrast, evidence demonstrates that patient-centered care, enhanced participation in medical decision-making, and effective self-management yield superior outcomes in improving cancer survivors’ quality of life compared to singular external support approaches^18,19^. However, Chinese HNC patients face distinct challenges in implementing these approaches. The disease predominantly affects middle-aged or elderly individuals and those with lower socioeconomic status^20^, who typically have limited health literacy^21^, impeding their ability to comprehend medical information and communicate healthcare needs effectively^22^. Moreover, deeply influenced by Chinese cultural traditions and healthcare systems^23,24^, these patients typically demonstrate high deference to medical professionals and passively accept medical decisions. Under these circumstances, patients’ limited engagement in their care may render “patient-centered care” superficial or even counterproductive, where increased provider efforts paradoxically result in heightened patient passivity and dependence. Therefore, enhancing HNC patients’ ability to voice for themselves is crucial for optimizing their healthcare outcomes.

Self-advocacy effectively addresses these challenges. It refers to patients’ confidence and ability to protect their interests when facing life-threatening diseases, encompassing skills like information seeking, question asking, expressing preferences, and protecting personal rights^25–27^. Research shows that patients with higher self-advocacy capabilities actively acquire disease knowledge, communicate effectively with healthcare providers, and participate in treatment decisions, improving treatment adherence and satisfaction^28,29^. Through self-advocacy, patients enhance self-care responsibility, adopt health-promoting behaviors, alleviate symptoms, and reduce psychological distress^30,31^. It also helps rebuild social roles, increase participation, and reduce isolation^32,33^.

The Patient Self-Advocacy Scale (PSAS) is one of the important tools for assessing this capability. It was developed by E. Brashers and Stephen M. Haas^25^ in 1999. Brashers validated the scale’s reliability and validity in samples of AIDS patients and general adults, with a Cronbach’s α coefficient of 0.78. The PSAS has demonstrated good applicability among patients in the United States and Iran^34,35^ but has not been translated into Chinese and validated. Therefore, this study aims to translate the PSAS into Chinese and conduct a psychometric evaluation using Classical Test Theory (CTT) and Item Response Theory (IRT) to provide a reliable instrument for assessing self-advocacy levels among Chinese HNC patients and potentially contributing to the improvement of patients’ quality of life.

## Methods

### Study design and sample

A cross-sectional study was conducted at the Department of Maxillofacial and Otolaryngological Oncology, Tianjin Medical University Cancer Institute and Hospital from November 2023 to August 2024. The inclusion criteria were: (1) histopathologically diagnosed with head and neck squamous cell carcinoma^1^ ; (2) clear consciousness with basic reading comprehension and verbal expression abilities sufficient to complete questionnaires; and (3) age ≥ 18 years. The exclusion criteria were: (1) non-primary head and neck cancer; (2) concurrent malignancies at other sites; and (3) psychiatric disorders or cognitive impairment. The exclusion criteria included: (1) voluntary withdrawal during the survey and (2) failure to complete the required survey responses for any reason.

According to Roscoe’s criterion, the sample size should be ten times the total number of items in the questionnaire^36,37^, with an additional 10% added to account for potential missing data. In this study, the questionnaire consisted of 12 items, resulting in a minimum required sample size of 132. However, considering that confirmatory factor analysis (CFA) requires a sample size greater than 200 ^38^ and that exploratory factor analysis (EFA) and CFA require different samples, a larger sample size was needed. Thus, to ensure more robust results, a total of 302 patients with HNC were recruited, and all collected questionnaires were deemed valid. The sample was randomly divided using a computer-generated sequence into two subsets: EFA (*n* = 102) and CFA (*n* = 200), while the complete dataset of 302 participants was utilized for all other analyses including item analysis, reliability testing, and IRT analyses. A total of 20 participants who voluntarily left contact information underwent a remeasurement after a 2-week interval to assess test-retest reliability.

### Instruments

#### Patient information form

A demographic and clinical information form was used to collect data including gender, age, educational level, marital status, employment status, place of residence, and living arrangements. Clinical data included cancer type, disease duration, and treatment modalities.

### The PSAS

The measurement instrument was developed by Brashers and Haas^25^. It consists of three dimensions: increased illness education, increased assertiveness in physician communication, and mindful nonadherence. The scale comprises 12 items rated on a 5-point Likert scale. Total scores range from 12 to 60, with higher scores indicating stronger self-advocacy capabilities. This questionnaire is self-administered by the patients.

### Translation and Cross-cultural adaptation of the PSAS

**Fig. 1:**
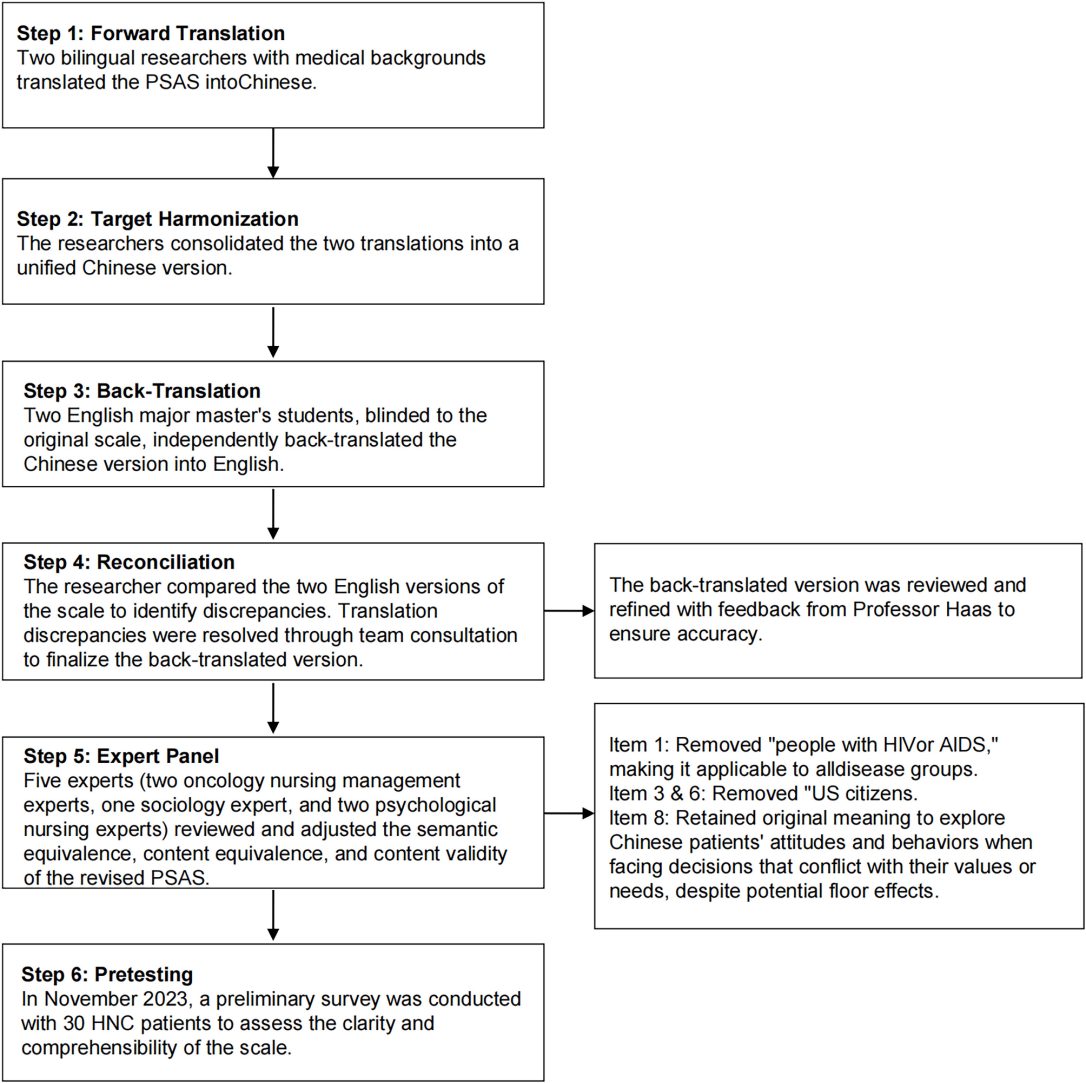
Translation and Cross-cultural Adaptation of the PSAS.

After obtaining permission from Professor Stephen M. Haas, we conducted the translation and cross-cultural adaptation following Brislin’s guidelines^39^ (Fig. 1). The majority of participants indicated that the questionnaire had an appropriate number of items, was easy to comprehend, and required 5–10 min to complete. Based on these results, the Chinese version of PSAS (PSAS-C) was finalized.

### Data analysis

#### Participant characteristics

Descriptive statistics were conducted and demographics between EFA and CFA groups were compared through Chi-square and rank-sum tests (*p* < 0.05) using SPSS 26.0.

### CTT-based analysis of psychometric properties

Statistical analyses were conducted using IBM SPSS Statistics version 26.0 and AMOS version 28.0. The psychometric evaluation included the following aspects: Item discrimination was evaluated through item-total correlation analysis, and items with correlation coefficients below 0.4 were eliminated^40^. The Critical Ratio method split participants into high-scoring (top 27%) and low-scoring (bottom 27%) groups based on total scores, with independent t-tests comparing groups (*p* < 0.05)^41^. For content validity, five experts rated item relevance on a 4-point Likert scale. The Item-level CVI (I-CVI) calculation involved the proportion of experts giving ratings of 3 or 4 per item, while Scale-level CVI (S-CVI) represented the average of all I-CVIs. Acceptable validity thresholds were I-CVI ≥ 0.78 and S-CVI/Ave ≥ 0.90 ^42,43^. Factor analysis appropriateness was assessed through the Kaiser-Meyer-Olkin measure (KMO > 0.6) and Bartlett’s test of sphericity (*p* < 0.01)^44^. The PSAS-C structural validity was evaluated using both EFA and CFA. EFA employed Principal Component Analysis with Promax rotation, requiring factor loadings ≥ 0.400 ^45^. CFA used maximum likelihood estimation, with acceptable model fit indices defined as χ^2^/df < 5, Standardized Root Mean Square Residual (SRMR) and Root Mean Square Error of Approximation (RMSEA) ≤ 0.08, and Incremental Fit Index (IFI), Tucker-Lewis Index (TLI), Comparative Fit Index (CFI) ≥ 0.90 ^43,46^. Reliability was evaluated through two approaches: internal consistency using Cronbach’s α (acceptable at ≥ 0.70) and test-retest reliability using the intraclass correlation coefficient (ICC) calculated with a two-way mixed-effects model with absolute agreement definition and single measurement (acceptable at ≥ 0.50)^47^.

### IRT-based analysis of psychometric properties

Model Selection: The Rasch model, as an IRT model, estimates response probability based on a person’s ability and item difficulty parameters on a logit scale. Unlike CTT, these parameters operate independently of other factors^48^. The Rating Scale Model (RSM)^49^, an extension of the Rasch model for Likert-type scales, provides parameter invariance, sample-independent item calibration, and item-specific measurement error estimation.

Rasch analysis was performed using Winsteps 3.6.6. The analysis tested structural validity by examining whether the data met the assumptions of unidimensionality and local independence, which was done using a statistical method called Principal Component Analysis of Residuals (PCAR). The first dimension needed to explain > 40% of the total variance, with the first contrast < 15% ^50^. Local independence was assessed by inter-item residual correlations (values < 0.7 indicating adequacy)^51^. Model fit adequacy used Outfit and Infit Mean Square values, with 0.50 to 1.50 indicating acceptable fit^52^, while point-measure correlations between 0.4 and 0.8 indicated acceptable item-dimension relationships. Measurement consistency was evaluated through reliability and separation indices. Person Reliability Index (PRI) and Item Reliability Index (IRI) assessed internal consistency, with values > 0.70 classified as acceptable^53^. Item Separation Index (ISI) and Person Separation Index (PSI) examined item difficulty hierarchy and respondent classification capacity, with values > 1.5 considered adequate^54^. Wright maps visualized person abilities and item difficulties distribution on the same logit scale. Differential Item Functioning (DIF) analysis examined item interpretation across demographic variables, including gender, age group, and long-term residence. DIF was considered significant when DIF contrast exceeded 0.5 logits, and the Mantel-Haenszel test showed *p* < 0.05 ^55^.

## Results

### Participant characteristics

The study included 302 participants with a mean age of 58.19 ± 8.30 years, comprising 227 males (75.2%) and 75 females (24.8%). The median PSAS-C total score was 34.00 (23.75, 42.00). The demographic characteristics of both the EFA and CFA groups are presented in Table 1. All the variables were comparable between both groups (*p* > 0.05).

**Table 1:** Participant characteristics.

Variables		EFA	CFA	*P*-value
		(*n* = 102)	(*n* = 200)	
Gender	Male	74(72.5%)	153(76.5%)	0.452
	Female	28(27.5%)	47(23.5%)	
Age	≤ 50 years	17(16.7%)	25(12.5%)	0.085
	51 ~ 70 years	69(67.6%)	158(79%)	
	>70 years	16(15.7%)	17(8.5%)	
Educational level	Primary school or below	26(25.5%)	41(20.5%)	0.614
	Junior high school	31(30.4%)	65(32.5%)	
	High school	31(30.4%)	68(34%)	
	Associate degree	6(5.9%)	23(11.5%)	
	Bachelor’s degree or above	8(7.80%)	3(1.5%)	
Marital status	Single	1(1.00%)	6(3%)	0.343
	Married	97(95.10%)	181(90.5%)	
	Divorced/Widowed	4(3.9%)	13(6.5%)	
Employment status	Employed	25(24.5%)	44(22%)	0.471
	Unemployed	45(44.1%)	103(51.5%)	
	Retired	32(31.4%)	53(26.5%)	
Permanent residence	Rural	44(43.1%)	81(40.5%)	0.372
	Township	9(8.8%)	29(14.5%)	
	Urban	49(48%)	90(45%)	
Living arrangements	Living alone	6(5.9%)	23(11.5%)	0.328
	Living with spouse	80(78.4%)	156(78%)	
	Living with spouse and children	14(13.7%)	18(9%)	
	Living with children	2(2%)	2(1%)	
	Other	0(0%)	1(0.5%)	
Cancer type	Sinus cancer	1(1%)	1(0.5%)	0.075
	Nasal cavity cancer	3(2.9%)	7(3.5%)	
	Laryngeal cancer	27(26.5%)	51(25.5%)	
	Oral cancer	43(42.2%)	107(53.5%)	
	Oropharyngeal cancer	5(4.9%)	14(7%)	
	Hypopharyngeal cancer	23(22.5%)	20(10%)	
Duration of illness	≤ 6 months	68(66.7%)	144(72%)	0.820
	7 ~ 12 months	16(15.7%)	26(13%)	
	13 ~ 48 months	15(14.7%)	25(12.5%)	
	≥ 49 months	3(2.9%)	5(2.5%)	
Current treatment method	Chemotherapy	43(42.2%)	86(42.5%)	0.534
	Radiotherapy	6(5.9%)	10(4.5%)	
	Targeted therapy	1(1%)	7(3.5%)	
	Surgery	46(41.2%)	91(44%)	
	Immunotherapy	6(5.9%)	6(3%)	

EFA: exploratory factor analysis.CFA: confirmatory factor analysis.

### Validity and reliability testing using CTT

#### Item analysis

Item-total correlations ranged from 0.776 to 0.942. CR values between high-scoring (top 27%) and low-scoring groups (bottom 27%) ranged from 13.269 to 33.170, with significant differences for all items (*p* < 0.05) (Table 2).

**Table 2:** Results of item analysis.

Item	*R*	CR
1	0.776	13.269
2	0.930	30.517
3	0.929	32.857
4	0.942	33.170
5	0.885	20.368
6	0.897	21.534
7	0.912	23.820
8	0.900	24.682
9	0.896	18.892
10	0.845	14.911
11	0.897	22.227
12	0.805	13.452

R = Item-total correlation, CR = Critical ratio. All differences between high and low groups were statistically significant (*p* < 0.001).

#### Content validity

The PSAS-C demonstrated item-level content validity index (I-CVI) values ranging from 0.80 to 1.00 and a scale-level content validity index (S-CVI) of 0.95.

#### Structural validity

The KMO value (0.908) and Bartlett’s test (χ^2^ =1239.048, *p* < 0.01) supported factor analysis. Exploratory factor analysis (EFA) was conducted using principal component analysis as the extraction method with oblique rotation, which identified three factors:: Increased illness education (Items 1–4), Increased assertiveness in physician communication (Items 5–7), and Mindful nonadherence (Items 8–12), with factor loadings ranging from 0.418 to 0.978 (Table 3).

**Table 3:** Exploratory factor analysis of the PSAS-C(*n* = 102).

Item	Factor 1	Factor 2	Factor 3
1. I believe it is important for me to learn as much as possible about my illnesses and treatments^a^	0.559	0.447	
2. I actively seek out information on my illnesses	0.938		
3. I am more educated about my health than most people	0.939		
4. I have full knowledge of the health problems of myself	0.961		
5. I don’t get what I need from my physician because I am not assertive enough.^b^		0.904	
6. I am more assertive about my health care needs than most people		0.918	
7. I frequently make suggestions to my physician about my health care needs		0.844	
8. If my physician prescribes something I don’t understand or agree with, I question it			0.418
9. Sometimes there are good reasons not to follow the advice of a physician			0.874
10. Sometimes I think I have a better grasp of what I need medically than my doctor does			0.978
11. If I am given a treatment by my physician that I don’t agree with, I am likely to not take it			0.846
12. I don’t always do what health care worker has asked me to do			0.885

^a^ Cross-loaded item.

^b^ Reverse-scored item.

**Fig. 2:**
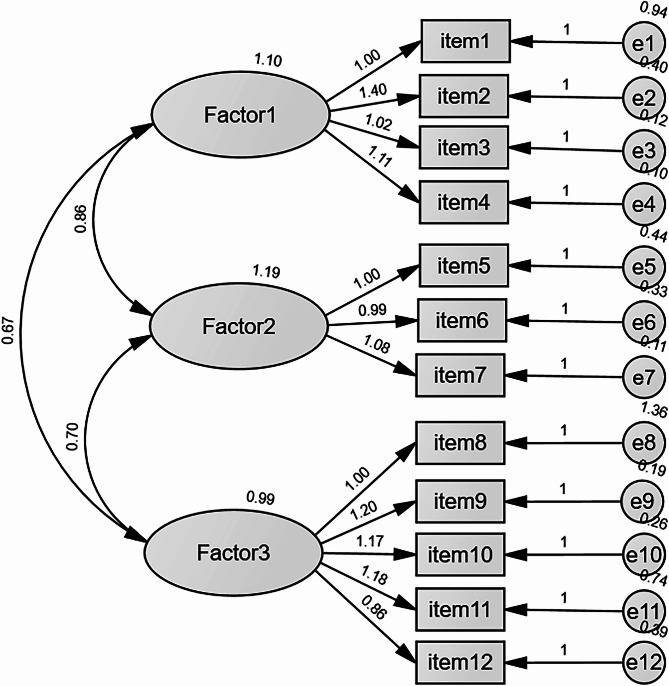
Confirmatory factor analysis model of the PSAS-C.

Confirmatory factor analysis of the three-factor model using maximum likelihood estimation showed the following fit indices: χ^2^/df = 2.595, RMSEA = 0.090, SRMR = 0.072, CFI = 0.966, IFI = 0.966, and TLI = 0.956. Although the RMSEA slightly exceeded 0.08, it remained acceptable (≤ 0.10). The standardized factor loadings for the three-factor model are illustrated in Fig. 2.

#### Reliability

The Cronbach’s α coefficient of the PSAS-C was 0.942 for the total scale and 0.922, 0.932, and 0.908 for the three dimensions. The two-week test-retest reliability showed an ICC of 0.840 for the total scale and 0.913, 0.703, and 0.815 for the three dimensions (Table 4).

**Table 4:** Results of ICC calculation using Single-Measurement, Absolute-Agreement, 2-Way Mixed-Effects model.

Dimensions	Intraclass Correlation	95% Confidence Interval		F Test With True Value 0			
		Lower Bound	Upper Bound	Value	df1	df2	Sig
Total score	0.840	0.605	0.936	13.715	19	19	0.000
Dimension 1	0.913	0.736	0.968	28.604	19	19	0.000
Dimension 2	0.703	0.345	0.875	6.983	19	19	0.000
Dimension 3	0.815	0.596	0.922	9.929	19	19	0.000

### Validity and reliability testing using IRT

#### Unidimensionality and local independence

The PSAS-C data met two key assumptions of Rasch analysis. Principal component analysis of standardized residuals showed that the first dimension extracted by the Rasch model explained 71.2% of the variance, with the first contrast accounting for 8.1%. Among the 66 item pairs, only Items 3 and 4 showed local dependency, with a correlation of 0.74 (> 0.70). This local dependency was theoretically explicable, as both items assessed patients’ cognitive awareness of their health status.

#### Rasch model fit

All items except Item 1 (Outfit MNSQ = 1.65, Infit MNSQ = 1.63) showed an excellent fit to the Rasch model. The point-measure correlations ranged from 0.68 to 0.83, indicating good item discrimination (Table 5).

**Table 5:** Rasch model analyses of the PSAS-C.

Item	Score(M ± SD)	MNSQ		Point-measure Correlation	
		Infit	Outfit	Corr.	ExP.
12	1.75(± 1.131)	1.13	0.92	0.68	0.68
10	1.90(± 1.276)	1.04	0.85	0.72	0.71
9	2.11(± 1.279)	0.79	0.77	0.77	0.73
11	2.30(± 1.478)	1.43	1.22	0.72	0.75
4	2.72(± 1.183)	0.63	0.77	0.80	0.77
3	2.84(± 1.119)	0.52	0.84	0.81	0.77
2	2.89(± 1.609)	1.17	1.05	0.78	0.78
8	3.10(± 1.499)	1.28	1.20	0.76	0.78
6	3.14(± 1.228)	0.83	0.91	0.80	0.78
7	3.45(± 1.221)	0.69	0.69	0.83	0.78
5	3.49(± 1.265)	0.83	0.91	0.80	0.78
1	3.90(± 1.384)	1.63	1.65	0.68	0.76

#### Reliability

The PSAS-C demonstrated an item reliability of 0.99 with a separation index of 12.98, indicating excellent consistency among items. The person reliability was 0.91 with a separation index of 3.16, suggesting the scale could effectively distinguish different levels of respondents.

#### Item measure and map

**Fig. 3:**
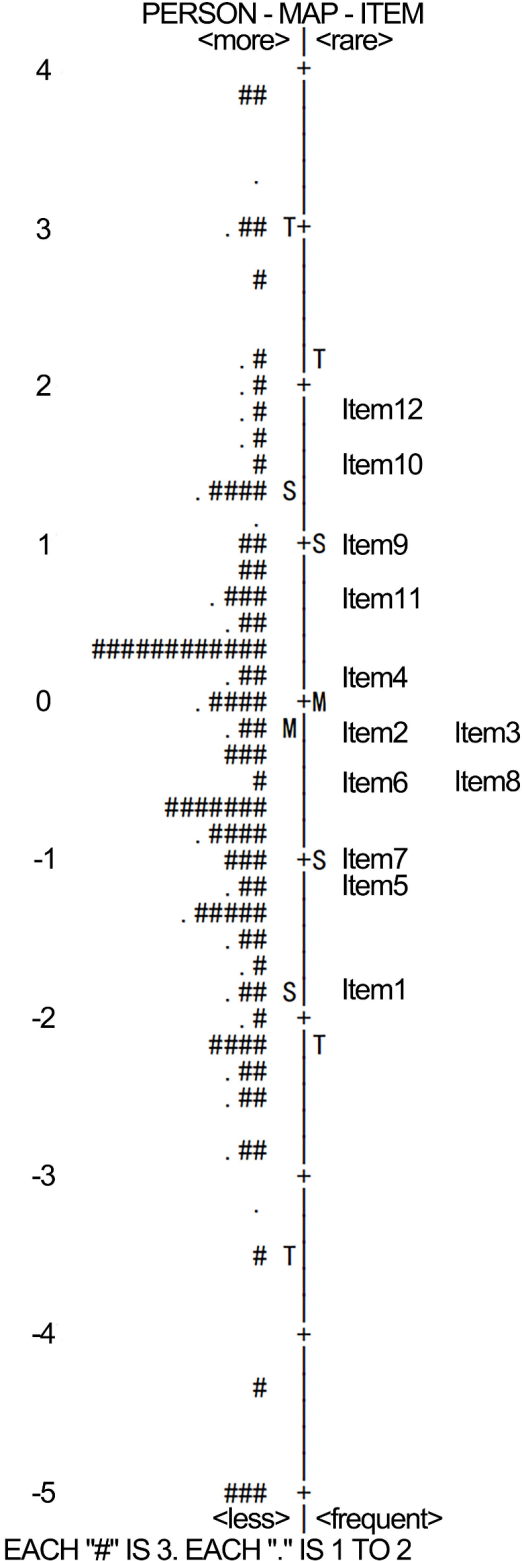
Wright Person-Item Rasch Map for the PSAS-C (*n* = 302). The Wright map demonstrates item difficulty and person ability distribution. The right side shows the distribution of items, with the most difficult items at the top and the least difficult at the bottom. The left side represents respondents′measured abilities, from the most capable at the top to the least capable at the bottom. The symbol “#” represents 3 persons, and “.” represents 1–2 persons.

Wright map showed that item difficulties ranged from − 2 to 2 logits with normal distribution. Person abilities ranged from − 5 to 4 logits. Mean item difficulty and person ability difference was < 1 logit, indicating good person-item matching (Fig. 3).

#### Differential item functioning

DIF analysis across genders, age groups, and long-term residence locations showed no significant differences. The absolute DIF contrast values ranged from 0.000 to 0.330 (p values ranged from 0.1588 to 0.9944) for gender, from 0.000 to 0.510 (p values ranged from 0.1408 to 0.9946) for age groups, and from 0.000 to 0.630 (p values ranged from 0.1320 to 0.9591) for residence locations. No items met the significance criteria (absolute DIF contrast > 0.5 logits and Mantel-Haenszel *p* < 0.05), indicating consistent item functioning across demographic groups for respondents with equivalent ability levels.

## Discussion

HNC patients face complex challenges, and patient self-advocacy provides a new perspective for improving their health outcomes. The application of the PSAS has the potential to realize patient self-management and patient-centered care concepts, thereby enhancing patients’ quality of life. This is the first PSAS validation study conducted in the East Asian region. The study employed both Classical Test Theory (CTT) and Item Response Theory (IRT), which represent two different but complementary measurement frameworks, to ensure robust psychometric properties.

The mean score of PSAS-C was 33.59 ± 12.32, which is lower than that reported in U.S. populations (39.63 ± 6.37)^34^. This discrepancy may be attributed to two characteristics of the U.S. cohort: a higher proportion of participants with advanced education (50.8% with a bachelor’s degree or above), which may enhance their ability to advocate for themselves, and the inclusion of breast cancer patients (30.8%). Compared to HNC patients, breast cancer patients typically have greater access to support services and educational resources.

Furthermore, in terms of psychometric evaluation results, the PSAS-C demonstrated excellent psychometric properties with high reliability, good content validity, and structural validity. Content validity indices (I-CVI and S-CVI/Ave) met recommended standards^42^. The scale showed higher internal consistency and test-retest reliability than previous American and Iranian studies^34,35^. Factor analyses confirmed the original three-factor structure. Before conducting Rasch analysis, we examined whether the scale satisfied unidimensionality and local independence assumptions. Interestingly, the total scale satisfied the unidimensionality assumption, and this difference stems from the two methods’ different theoretical foundations and assumptions rather than indicating problems with the scale’s structural validity and stability. Through Rasch analysis, the PSAS-C showed a good fit except for Item 1’s insufficient fit, which assesses patients’ basic attitudes toward understanding illness and treatment plans. As most patients agree with this view, the item poorly differentiates self-advocacy levels. This misfit is acceptable, given its conceptual importance and other psychometric properties. The scale showed reasonable difficulty distribution and measurement fairness across demographic groups.

Beyond the basic psychometric properties, notable differences emerged in the factor structure: Item 8 (“If my physician prescribes something I don’t understand or agree with, I question it”) shifted from Factor 2 to Factor 3. This change might reflect China’s unique healthcare context. With the progression of healthcare system marketization, patients’ perception of healthcare providers has become discordant, creating conflict between their traditional implicit reverence for medical professionals and providers’ “profit-driven image”^23,24^. During self-advocacy, patients rarely question medical orders from a professional perspective due to respect for medical authority but demonstrate higher sensitivity and willingness to question when medical costs are involved. This suggests that patients’ motivation to question medical orders stems more from economic considerations than confidence levels, which warrants careful interpretation of Item 8 in practical applications of the scale.

The main limitations of this study include single-hospital recruitment affecting sample representativeness and the absence of a gold standard for self-advocacy assessment in China preventing criterion-related validity analysis. Future studies should address these limitations through multi-center recruitment and establishing criterion validity.

Future research needs to address several key challenges to promote patient self-advocacy. First, assessing healthcare providers’ acceptance of patient self-advocacy and establishing provider-patient trust, as healthcare providers may resist patient self-advocacy due to concerns about consultation time extension and perceived threats to their professional authority^25^. Second, it is essential to longitudinally evaluate patient self-advocacy across disease trajectories, as patients’ advocacy needs and capabilities vary at different stages of illness. For example, newly diagnosed patients often struggle with medical decision-making due to the complexity of information and emotional distress, whereas patients with advanced or recurrent cancer may encounter additional challenges, such as symptom burden or diminished confidence in their ability to self-advocate^31^. Third, exploring strategies to balance individual advocacy with family-centered decision-making in the context of traditional values. Chinese culture emphasizes family as the core social unit where members are interdependent and share responsibilities^56^. Family members serve as key stakeholders with deep involvement in major medical decisions. Although this familial model may lead to lower PSAS scores, it does not necessarily reflect patients’ lack of willingness or capacity for self-advocacy.

## Conclusion

The PSAS-C demonstrates good validity, reliability, and objectivity. The scale shows good operability and feasibility with its concise items, simple scoring method, and moderate difficulty level. It accurately measures self-advocacy levels among Chinese HNC patients, providing healthcare providers with a scientific assessment tool.

## Data Availability

The datasets generated and/or analysed during the current study are not publicly available due to privacy concerns and confidentiality agreements with study participants but are available from the corresponding author on reasonable request.

## Abbreviations

CFA: Confirmatory factor analysis
CFI: Comparative fit index
CR: Critical ratio
CTT: Classical test theory
CVI: Content validity index
DIF: Differential item functioning
EFA: Exploratory factor analysis
HNC: Head and neck cancer
ICC: Intraclass correlation coefficient
IFI: Incremental fit index
IRI: Item reliability index
IRT: Item response theory
ISI: Item separation index
KMO: Kaiser–Meyer–Olkin measure
MNSQ: Mean square
PCAR: Principal component analysis of residuals
PRI: Person reliability index
PSAS: Patient Self-Advocacy Scale
PSAS-C: Chinese Version of the Patient Self-Advocacy Scale
PSI: Person separation index
RSM: Rating scale model
RMSEA: Root mean square error of approximation
SRMR: Standardized root mean square residual
S-CVI: Scale-level content validity index
TLI: Tucker-Lewis index
